# Distalization of Maxillary First Permanent Molar by Pendulum Appliance in Mixed Dentition Period

**DOI:** 10.5005/jp-journals-10005-1454

**Published:** 2017-02-27

**Authors:** Sujatha Paranna, Prakashchandra Shetty, Latha Anandakrishna, Anuradha Rawat

**Affiliations:** 1 Senior Lecturer, Department of Pediatric Dentistry, P.M. Nadagouda Memorial Dental College and Hospital, Bagalkot, Karnataka, India; 2 Professor, Department of Pedodontics and Preventive Dentistry, Faculty of Dental Sciences, M.S. Ramaiah University of Applied Sciences, Bengaluru, Karnataka, India; 3 Professor and Head, Department of Pedodontics and Preventive Dentistry, Faculty of Dental Sciences, M.S. Ramaiah University of Applied Sciences, Bengaluru, Karnataka, India; 4 Senior Lecturer, Department of Orthodontics, J.K.K. Nattaraja Dental College & Hospital, Namakkal, Tamil Nadu, India

**Keywords:** Distalization, Interceptive orthodontics, Maxillary molar, Pendulum appliance.

## Abstract

**Introduction:**

Mesial drifting of molar teeth in maxillary arch is corrected by movement of the molars distally. In addition to traditional distal movement techniques, such as extraoral force application and removable appliances, various intra-arch devices have been introduced since 1980s. These intra-arch appliances have nearly eliminated the need for patient cooperation.

**Case report:**

The purpose of this paper is to report a case of 10-year-old male patient with loss of space in maxillary molar teeth treated by intra-arch appliance-pendulum appliance by distalization of maxillary first permanent molar teeth. Distaliza-tion of the permanent molar teeth helped in proper eruption of second premolar teeth without any extensive treatment procedures.

**Conclusion:**

In the present case report, the treatment of developing malocclusion was corrected by utilizing the concept of interceptive orthodontics. Hence, correction of space loss in mixed dentition period using pendulum appliance can eliminate the fixed orthodontic therapy.

**How to cite this article:**

Paranna S, Shetty P, Anandakrishna L, Rawat A. Distalization of Maxillary First Permanent Molar by Pendulum Appliance in Mixed Dentition Period. Int J Clin Pediatr Dent 2017;10(3):299-301.

## INTRODUCTION

Most traditional approaches to molar distalization include extraoral traction, Wilson distalizing arches, removable spring appliances, and intermaxillary elastics with slidingjigs, which require considerable patient compliance to be successful. More recently, the subjectivity and problems of predicting patient behavior have led many clinicians to devise appliances that minimized reliance on the patient and that are under the control of the clinician.^1-3^

Another popular method of molar distalization that requires no cooperation is the so-called "pendulum" appliance system.^4^ Hilgers^5^ of California introduced the "Pendulum Appliance" in 1992 as a mechanism for class II noncompliance treatment. The pendulum appliance uses a large Nance acrylic button for palatal anchorage and 0.032" titanium-molybdenum alloy (TMA) springs to deliver a light, continuous force to the upper first molars without affecting the palatal button. The appliance produces a broad pendulum of force from the midpalate to the upper molars.^6^

Hilgers^5^ stated that it is typical to see approximately 5 mm of distal molar movement in a 3 to 4 months period of time and that 20% of the space opening can be ascribed to anterior anchorage loss.

The purpose of this case report is to describe the correction of space deficiency by distalization of maxillary permanent first molars using pendulum appliance.

## CASE REPORT

**Fig. 1:**
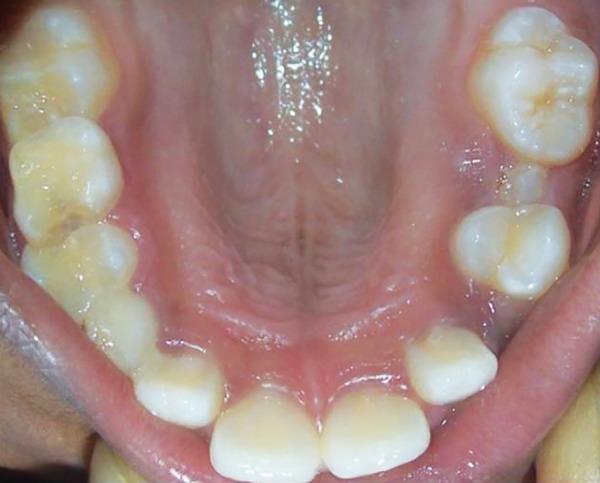
Preoperative intraoral view with space deficiency for eruption of 25

**Fig. 2:**
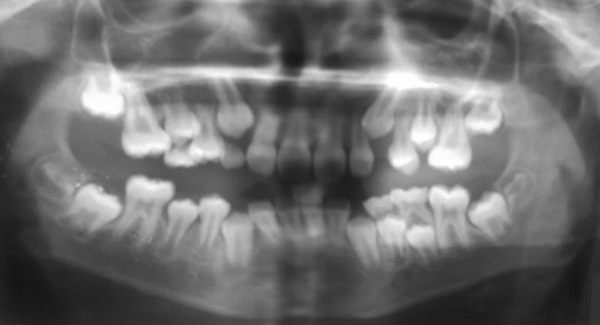
Preoperative orthopantomogram with space deficiency for eruption of both 25 and 15

**Fig. 3:**
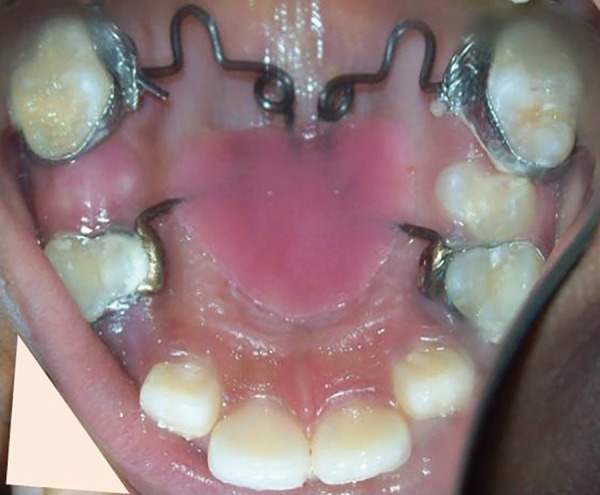
Pendulum appliance cemented with bands on 14, 16, 26, and 24

A healthy 10-year-old male patient presented to the Department of Pediatric and Preventive Dentistry, M.S. Ramaiah Faculty of Dental Sciences, Bengaluru, Karnataka, India, for routine dental care. Oral examination revealed mixed dentition, with deficient space for eruption of second premolar teeth (Figs 1 and 2). The treatment planned was distalization of maxillary first permanent molars by intra-arch pendulum appliance developed by Hilgers. Both the maxillary first permanent molars and first premolars were banded and impression of maxillary arch was made. The pendulum appliance design consisted of anterior acrylic nance portion with two posteriorly extending TMA coil springs made of 0.032". The plane of the coil springs should be parallel to the maxillary plane; the extensions of TMA wire are then soldered to the molar and premolar bands (Fig. 3). The appliance was cemented onto the molars and premolars. The appliance was activated extraorally and was cemented. The appliance was monitored at monthly intervals and the appliance was removed for reactivation and recementation.

**Fig. 4:**
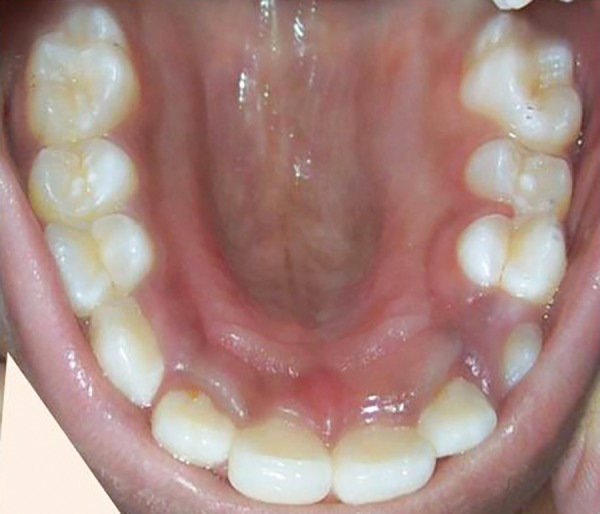
Postoperative intraoral photograph with eruption of 15 and 25 in proper alignment

**Fig. 5:**
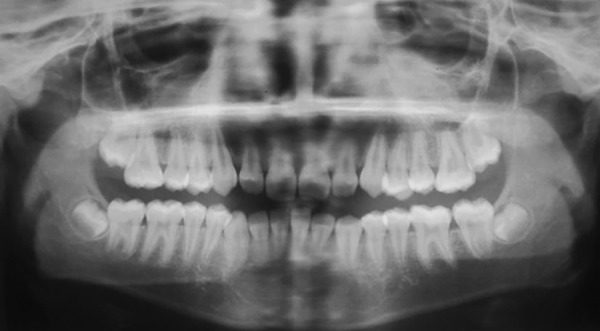
Postoperative radiograph with proper alignment of teeth

At the end of 4th month sufficient space was regained and second premolars started erupting into the space gained. At 7th month with the complete eruption of premolars and canines proper maxillary arch alignment was achieved (Figs 4 and 5). The results of this case study have shown that the pendulum appliance is an effective and reliable method for distalization of maxillary molar teeth.

## DISCUSSION

Distal movement of the molars appears to be more efficient before the eruption of the upper second molars.^7^ In the present case report, only the first permanent molars were erupted, which indicates the best time for distaliza-tion of molars using pendulum appliance.

The indications for the pendulum appliance are:

- First phase of orthodontic treatment for unilateral or bilateral distalization of maxillary first molar teeth for correction of Class II molar relationship in noncompli-ant patients.
- Space regaining in cases of mesial drift of upper first molars due to early loss of primary molars; and
- Nonextraction treatment of mild-to-moderate crowding.^8^

The major advantages of the appliance lie in its minimal dependence on patient compliance, ease of fabrication of appliance, allow correction of minor transverse and vertical molar positions by adjustment of the springs, and last but not the least patient-acceptance.^8^

## LIMITATIONS OF PENDULUM APPLIANCE

- Torquing or rotation of molars: If the helix loop is not adjusted correctly, the pendulum spring can be distorted and can result in undesirable rotation or torquing of the molars.^9^
- Tissue irritation:
  – Food and plaque accumulation under the palatal acrylic causes slight tissue inflammation. This does not limit the use of this appliance.
  – The activated helix loop of the pendulum springs causes anterior reciprocal forces to be generated against the palatal acrylic and the palate. With a larger palatal acrylic, the generated forces are spread over a wider area with minimal palatal irritation.

## CONCLUSION

Patient tolerance of the pendulum appliance is excellent. It is simple and easy to fabricate, with minimal laboratory support. The cost of a pendulum appliance is a fraction of the cost of commercially available molar distalization appliances.
